# Supplementary material for: Radiopharmaceuticals for PET and SPECT Imaging: A Literature Review over the Last Decade
Source: Int J Mol Sci. 2022 Apr 30;23(9):5023. doi: 10.3390/ijms23095023 (PMC9103893; doi:10.3390/ijms23095023)
Supplement: Supplementary file 1 [file ijms-23-05023-s001.zip › ijms-1689207-supplementary.pdf]

Supplementary Material

Radiopharmaceuticals for PET and SPECT Imaging: A literature review over the last decade

George Crișan[1,2], Nastasia Sanda Moldovean-Cioroianu[1], Diana-Gabriela Timaru[1], Gabriel Andrieș[2], Călin Căinap[3], and Vasile Chiș[1,4,*]

[1] Faculty of Physics, Babeş-Bolyai University, Str. M. Kogălniceanu 1, RO-400084 Cluj-Napoca, Romania
[2] Department of Nuclear Medicine, County Clinical Hospital, Clinicilor 3-5, Cluj-Napoca 40 0 0 06, Romania
[3] The Oncology Institute "Prof. Dr. Ion Chiricuţă", Republicii 34-36, 400015 Cluj-Napoca, Romania
[4] Institute for Research, Development and Innovation in Applied Natural Sciences, Babeș-Bolyai University, Str. Fântânele 30, RO-400327 Cluj-Napoca, Romania
* Correspondence: vasile.chis@ubbcluj.ro

Table S1. PET Radiopharmaceuticals used in oncology (references over the last ten years)

Radiotracer	Disease	Molecular target	Function	Properties	Ref.
[methyl-^{11}C]methionine	urinary, gynecological, liver and lung cancer	L-type amino acid transporter system and Na+-dependent system	imaging the rate of protein synthesis	the short half-life of [^{11}C] limits the accessibility for PET scannings; [^{11}C]MET has been also widely used in various brain tumors	[3], [17], [48] [49], [52-59], [500]
[^{11}C]CO	wide applications in clinical research	a variety of chemotypes (amides, ketones, acids, esters, and ureas)	the production of a wide range of drug-like molecules and radioligands	require the presence of transition metals (e.g. Pd) as reagents; poor solubility of in organic solvents and high dilution in inert gas	[48], [55]
[^{11}C]acetate	prostate cancer, hepatocellular carcinoma, lung cancer, nasopharyngeal carcinoma, renal cell carcinoma, bladder carcinoma and brain tumors	all over the body	tracer for cytoplasmic lipid synthesis (increased in tumours); measurements of myocardial oxygen consumption	acetate is a molecule recruited by cells to convert into acetyl-CoA by acetyl-CoA synthetase; rapidly picked-up by cells; originally employed in cardiology; salt vector	[60], [61-71]
[^{11}C]erlotinib	cell lung carcinoma; colorectal cancer	epidermal growth factor receptor	tracing specific binding for activating mutations of the EGFR kinase	small molecule vector; has the structure identical to the clinically used drug; [^{11}C]-erlotinib	[4], [72-74]

			domain	accumulates in NSCLC tumors with activating kinase domain mutations	
[^{11}C]PD153035	cell lung carcinoma; colorectal cancer	epidermal growth factor receptor	quantify EGFR expression	small molecule vector; large differences of the uptake levels in tumors	[4], [445]
[^{11}C]choline	prostate cancer	phospholipid synthesis	tumor imaging; diagnostic agent	salt vector; as the proliferation of cancer cells gets higher, tumor cells exhibit an increased rate of the radiotracer’s uptake	[4], [59]
[^{18}F]F-choline	prostate cancer	phospholipid synthesis	primary staging, biochemical recurrence	salt vector; greater accuracy when compared to [^{18}F]FDG	[32], [398], [399]
[^{18}F]FCH	breast, thyroid, lung, brain, liver, prostate cancers	cellular membrane phospholipids, primarily in cancers	tumor imaging agent for various types of tumors	better than [^{18}F]FDG for PC and HCC detections; enters the cells through choline transporters, with accumulation in tumors due to malignancy-induced overexpression of choline kinase (CK)	[4], [399]
[^{18}F]FDOPA	glioma, neuroendocrine tumors, prostate cancer	amino acid transport; a multiple-target molecule	image a large variety of neuroendocrine tumors and pancreatic beta-cell hyperplasia	amino acid vector; good modality for detection of persistent and residual medullary thyroid cancer	[4], [197]
[^{18}F]FDG	neoplasm	glucose metabolism	tracer used for detection, staging and management of many types of cancer	[^{18}F]-FDG avidly accumulates in poorly proliferating and hypoxic cancer cells, but low in proliferating cancer cells	[17], [27],[33], [35], [38], [61], [117], [373], [390] [459-461], [500]
[^{18}F]FLT	solid malignancies; multiple tumors (breast, rectal, gastric, pancreatic, oesophageal	all over the body; thymidine kinase	tumor detection, staging, restaging, and response assessment to treatment, and	nucleoside vector; has been introduced for imaging tumor cell proliferation; it is monophosphorylated by thymidine kinase 1	[19], [20], [27], [400]

	cancers, non-Hodgkin lymphoma)		visualises cellular proliferation	(TK1), which leads to intracellular trapping; able to evaluate tumor heterogeneity, DNA replication	
[^{18}F]FMISO	neck, lung, breast, brain pancreatic, cervical cancer	various target localizations of tumor hypoxia	determining the duration of survival without relapses ; predicting the radiotherapy efficiency malignant tumors;	the most established agent for assessing hypoxia and has been used for cancer imaging over the past 30 years for glioblastoma multiforme, non-small-cell lung cancer, and head and neck tumors	[4], [22], [23], [29], [30]
[^{18}F]NaF	metastatic stages of various *cancers* and predictor of *myocardial* infarction	N.A.	detection of benign and malignant osseous abnormalities; bone remodeling; also used for the assessment of active calcification processes in coronary artery disease	it represents the bone-seeking PET radiotracer; considered as an excellent substitute for traditionally used 99mTc-labeled tracers; has favorable pharmacokinetics: high bone uptake, minimal binding to serum proteins, and fast clearance from the soft tissues	[4], [31]
[^{18}F]FGln	glioma, prostate, breast and thyroid cancer	glutamine-targeting metabolic tracer	particularly suitable for intracranial gliomas imaging	testing its ability to image other types of cancers is ongoing	[4]
[^{18}F]FSPG	hepatocellular carcinomas, intracranial malignancies, head and neck cancers, colorectal cancer, non-Hodgkin lymphoma	taken up by cells through the system xC−transporter *	detection and diagnostic tracer; high uptake in both small animal and human studies of intracranial malignancies	has the ability to identify drug-resistance by detecting upregulated antioxidant pathways and provides indicators of tumor response to treatment;	[4], [16]
[^{18}F]FACBC [^{18}F]FACPC	prostate carcinoma, breast cancer	increased flow into tumor cells *via* amino acid transporters: alanine-serine-cysteine transporter 2 (ASCT2) and large neutral amino acid transporter (LAT-1)	prediction of prostate cancer aggressiveness; breast tumor imaging for detection of locoregional and distant spread;	greater activity in malignant versus benign etiology; aminoacid vectors; diagnostic assessment and neurosurgery of gliomas	[4], [16]

Supplementary Material

[^{18}F]FDHT	prostate cancer	androgen receptors	a promising prognostic and diagnostic/predictive biomarker	hormone vector; can identify increased AR expression in high-grade glioma;	[4], [21], [23]
[^{18}F]ML-10<br>[^{18}F]ICMT-11	glioblastoma multiforme, breast cancer, lung cancer	apoptotic cells	assessment of apoptosis early after treatment	small molecule vectors;<br>the apoptosis response of intracranial tumor	[4]
[^{18}F]BMS-986192	cell lung carcinoma	programmed death-ligand 1	quantify PD-L1 expression in non–small-cell lung cancer	adnectin vector	[149],[342], [365]
[^{18}F]PSMA-1007<br>[^{18}F]DCFPyL<br>[^{18}F]rhPSMA-7	prostate cancer	prostate-specific membrane antigen	bone and soft tissue metastases from prostate cancer	peptidomimetic vectors;<br>reliably detects malignant lymph nodes and has an exceptional specificity of more than 99% for nodal metastases	[4], [32], [99]
[^{18}F]PARPi	head and neck cancer	poly(ADP-ribose) polymerase 1	DNA repair marker;<br>it accumulates mostly in the nuclei of tumor cells	small molecule vector;<br>it provides high-contrast images compared to [^{18}F]FDG	[39-42]
[^{18}F]FP-R01-MG-F2<br>[^{18}F]F-αvβ6-BP	head and neck cancer, lung, colorectal, breast, pancreatic	integrin αvβ6	targeting the cancer-associated integrin αvβ6	significant uptake in both the primary lesion and metastases, including metastasis to brain, bone, liver and lung	[4]
[^{18}F]F-Galacto-RGD<br>[^{18}F]F-RGD-K5<br>[^{18}F]F-fluciclatide<br>Al[^{18}F]F-alfatide-I<br>Al[^{18}F]F-alfatide-II	solid malignancies;<br><br>across multiple tumor types	integrin αvβ3 expressed by macrophages and angiogenic endothelial cells	markers of plaque inflammation and, potentially, of plaque vulnerability	peptide vectors;<br>tracers binding specifically to αvβ3	[4]
[^{18}F]EF5<br>[^{18}F]FAZA<br>[^{18}F]HX4		target the mitochondrial complex I and intratumor oxygen levels	detection of hypoxia;	small molecule vectors;<br>can predict the response of tumors to single fraction radiation treatment	[22-29]
[^{18}F]-BAY864367	prostate cancer, breast cancer, glioma	gastrin-releasing peptide receptor	gastrin-releasing-peptide receptor imaging	peptide vector;<br>bombesin analogue tracer	[4], [23]
[^{18}F]FDGal	hepatocarcinoma	galactose	N.A.	small molecule vector	[4],

		metabolism			[33-35]
[^{18}F]afatinib	lung carcinoma colorectal cancer	epidermal growth factor receptor	detection of EGFR positive tumors	small molecule	[4], [117]
[^{124}I]I-codrituzumab	hepatocarcinoma	glypican 3	detects tumor localization in most patients with HCC	antibody vector	[4], [75], [111]
[^{124}I]I-girentuximab	renal cell carcinoma	carbonic anhydrase 9	discriminates between clear-cell RCC (ccRCC) and non-ccRCC	antibody vector	[97]
[^{124}I]I-huA33	colorectal cancer	specific to A33 glycoproteins	N.A.	antibody vector	[4], [92]
[^{64}Cu]Cu-plerixafor	hematological and solid malignancies	C-X-C chemokine receptor type 4	N.A.	small molecule	[166]
[^{64}Cu]Cu-CB-TE2A-AR06	prostate and breast cancer	gastrin-releasing peptide receptor	N.A.	peptide vector	[165-167]
[^{64}Cu]Cu-ATSM	solid malignancies	tumor hypoxia and prognosis	imaging agent targeting the hypoxic regions in tumors	small molecule; uptake region of tumors associated with upregulation of DNA repair	[166], [168-170], [171], [174], [175]
[^{64}Cu]-PSMA	prostate cancer	prostate-specific membrane antigen	diagnostic and assessment of residual disease	comparable detection rates with [^{18}F]-PSMA	[4], [164]
[^{64}Cu]-DOTA-AE105	breast, lung, colorectal, prostate and bladder cancer	a promising uPAR-PET ligand in several preclinical validation studies; peptide antagonists AE105	diagnostic/imagi ng; prognostic in cancer invasion and metastasis	first in-human use in 2013	[4], [166]
[^{68}Ga]citrate	prosthetic joint/bone infections,	N.A.	diagnosis of bone infection	^{68}Ga-citrate presents many advantages over ^{67}Ga for the diagnosis of bone infections	[4]
[^{68}Ga]Ga-DOTA-SP	glioma	neurokinin 1 receptor	N.A.	peptide vector	[4]
[^{68}Ga]Ga-NOTA-exendin-4	insulinoma	glucagon-like peptide 1 receptor	detection of increased GLP-1R expressions;	also feasible in patients with multiple endocrine neoplasia	[177], [181]
[^{68}Ga]Ga-NOTA-Aca-BBN	prostate cancer, breast cancer, glioma	gastrin-releasing peptide receptor	for early tumorigenesis		[183]
[^{68}Ga]Ga-HER2-nanobody	breast cancer	epidermal growth factor receptor 2	nanobody directed against	nanobody vector	[184]

			HER2		
[68Ga]Ga-FAPI-04<br>[68Ga]Ga-FAPI-21<br>[68Ga]Ga-FAPI-46	solid malignancies	fibroblast activation protein α	quinoline-based PET tracers; fibroblast-activation-protein inhibitors	small molecule vectors	[4]
[68Ga]Ga-ABY-025	breast cancer	epidermal growth factor receptor 2	allows for the determination of HER2 expression heterogeneity	affibody vector; overcomes disadvantages of samples from biopsies	[184]
[68Ga]Ga-NeoBOMB1<br>[68Ga]Ga-BBN-RGD<br>[68Ga]Ga-RM26<br>[68Ga]Ga-SB3<br>[68Ga]Ga-RM2	prostate and breast cancer,<br>glioma	gastrin-releasing peptide receptor	N.A.	peptide vectors	[4], [182]
[68Ga]PSMA-I&T<br>[68Ga]PSMA-617<br>[68Ga]PSMA-11	prostate cancer	prostate-specific membrane antigen	for increased PSMA expression	peptidomimetic vectors: small proteins designed to mimic a peptide	[10], [179]
[89Zr]Zr-DFO-HuMab-5B1	pancreatic and bladder cancer	CA19-9 positive malignancies	accumulation of HuMab-5B1 in CA19-9 expressing tumors	antibody vectors;<br><br>several of these tracers might provide individualized antibody-based treatment;<br><br>despite their specificity, efficacy has remained limited	[90], [132]
[89Zr]Zr-DFO-MSTP2109A	prostate cancer	transmembrane epithelial antigen of prostate-1	a humanized IgG1 monoclonal antibody directed against STEAP1		[4], [113], [131], [153]
[89Zr]Zr-girentuximab	renal cell carcinoma	N.A.	differentiation between ccRCC and non-ccRCC lesions		[141]
[89Zr]Zr-bevacizumab	solid malignancies; particularly for malignant breast lesions	vascular endothelial growth factor receptor	early detection; VEGF-A overexpression		[4], [113], [118], [119], [121], [124], [145]
[89Zr]Zr-atezolizumab	cell lung carcinoma, bladder cancer, breast cancer	programmed death-ligand 1	clinical response to PD-L1 blockade in cancer		[4], [134]

[89Zr]Zr-cetuximab [89Zr]Zr-panitumumab	lung carcinoma, colorectal cancer	epidermal growth factor receptor	potential predictive biomarker for dosing strategy; novel immuno-PET tracers		[4], [113], [127], [128], [116], [138], [140]
[89Zr]Zr-ipilimumab	metastatic melanoma	cytotoxic T-lymphocyte-associated protein 4	monoclonal antibody targeting CTLA-4		[4], [113], [161]
[89Zr]Zr-Df-IAB22M2C	melanoma, lung cancer, hepatocarcinoma	CD8, CD20	noninvasive imaging of CD8+ T cells		[113], [151]
[89Zr]Zr-GSK2849330	solid malignancies	epidermal growth factor receptor 3	target engagement of mAb to the HER3 receptor		[4], [146]
[89Zr]Zr-rituximab [89Zr]Zr-obinutuzumab	B cell lymphoma	CD8, CD20	noninvasively and quantitatively monitor CD20/CD8-expression; monitor lymph node and peribronchial increased activity		[4], [113], [129]
[89Zr]Zr-AMG 211	gastrointestinal adenocarcinoma	carcinoembryonic antigen	accumulation in CD3-rich lymphoid tissues		[4], [137]
[89Zr]Zr-HuJ591	(advanced metastatic) prostate cancer	prostate-specific membrane antigen	PSMA overexpression and localization of disease		[103], [113]
[89Zr]Zr-MMOT0530A	pancreatic ductal adenocarcinoma and ovarian cancer	mesothelin **-tumor differentiation antigen	uptake in primary and metastatic PC and OC tumor lesions		[4], [113],[133]

* an amino acid antiporter that mediates the exchange of extracellular cystine and intracellular glutamate across the cellular membrane;
** frequently overexpressed in pancreatic and ovarian cancers
N.A. not applicable or not available.

Table S2. PET Radiopharmaceuticals used in neurology (references over the last ten years)

Radiotracer	Disease/ Molecular target	Function	Properties	Ref.
[11C]MET	brain gliomas and metastases	differentiation of tumor regrowth; delineation of gliomas.	the tracer's stability in its final formulation is not well documented in literature	[272]
N-[11C]-methyl-flumazenil	neuronal damages, epilepsy, stroke-induced penumbral, infarction and AD	binds to the benzodiazepine sites of GABAA receptors	excellent kinetic properties for image quantification	[272]
[11C]raclopride	psychiatric, PD, addiction, attention-deficit hyperactivity disorder, schizophrenia	tracer for dopamine function in striatal cortex	most widely used PET radiotracer for measuring DA changes in dopamine rates at the synaptic level	[272]
[11C]UCB-J	targeting synaptic vesicle proteins SV2A; primary interest in epilepsy, or diseases associated with synaptic loss	imaging SV2A expression in synaptic vesicles	leading SV2A tracer; good selectivity, fast kinetics	[275-280]
[11C]BU99008	targeting the imidazoline receptors I2BS; powerful tool in drug development and studies of neuropsychiatric disorders	imaging of 12BS distribution across the brain	the only tracer designed for I2BS in humans; good response in blocking studies; requires long scan times	[281-284]
[11C]ITMM	in cerebellar cortex; white matter<br><br>PD, addiction, epilepsy, neuropathic pain, and depression	targeting metabotropic glutamate receptors mGluR1	very good in vivo block responses, differences in cerebellar ataxia patients; low brain uptake and slow kinetics	[285-291]
[11C]LY2795050	AD, depression and addiction	targeting opioid receptors *k*	good pharmacokinetics for imaging; good response for *in vivo* blocking; limited dynamic range	[292-294]
[11C]Cimbi-36	AD, obsessive-compulsive disorder, Tourette's syndrome, schizophrenia	targeting serotonine 5-HT2; high uptake in cortical regions	good response to in vivo blocking; moderately slow kinetics	[295-298]
[11C]MK-3168	pain, addiction, and Tourette syndrome	fatty acid amide hydrolase associated receptors	slow kinetics and rapid metabolism in humans	[312-314]
[11C]Martinostat	schizophrenia; cerebellum uptake;	CNS quantification of HDACs	high brain uptake	[308-311]

[11C]PS13	dysfunctions of enzymes within the CNS	imaging COX-1	limited data available; low plasma free fraction, suitable kinetic profile	[299], [300], [322]
[11C]IMA-107	HD, PD and schizophrenia	study of PDE10A	good selectivity; good response in HD patients, suitable pharmacokinetics	[272]
[11C]Lu AE92686	HD, schizophrenia	N.A.	high brain penetration	[272]
[11C]CURB (URB694)	targeting distribution of FAAH; high uptake in the cerebral cortex, cerebellum, and hippocampus	FAAH tracer; promising pre-clinical blocking profiles in pre-clinical models and humans	very good response in blocking studies; irreversible kinetics	[272]
[18F]FET	brain tumor; does not serve as substrate to protein synthesis; glioma18F-FET uptake is not significantly influenced by changes in the BBB permeability	good diagnostic performance; highly specific for glioma	evaluation of its applicability in non-clinical research is still lacking; overcomes known limitations of [18F]FDG: increased uptake in inflammatory environment and elevated background signal in normal brain	[17]
[18F]AV-1451 most published *tau* tracer	tau proteins (tauopathies), AD	AD assessment; distinguishes between disease stages	high selectivity over amyloid; fast kinetics	[217], [219], [223], [232],[237]
[18F]THK 523, 5117,5317, 5351	uptake in the cerebellar cortex, neocortex, subcortical white matter, cerebellar grey matter	distinguishes AD from healthy controls	only moderate selectivity over amyloids, high white matter retention; limited selectivity reported data	[239-243], [247], [250], [272]
[18F]MK-6240	AD	imaging neurofibrillary tangles	low bindings in healthy controls; strong correlation with cognitive AD scores	[221], [318]
[18F]PM-PBB3	AD, cerebral accumulations of tau deposits	tau imaging	lower binding in basal ganglia and thalamus when compared to [11C]-PBB3	[272], [253]
[18F]FEOBV	targeting cholinergic system; cerebellar grey matter, and striatum	acetylcholine transporters and cholinergic synapses; studying degenerative conditions	improved signal-to-noise over previous VACht tracers; but slow kinetics	[268], [272]
[18F]MNI-444	targeting adenosine system A_{2A}; cerebellum	novel PET radiotracer for imaging A2A	high selectivity; good pharmacokinetics; low background, slow	[273], [274], [311]

			metabolism	
[18F]UCB-H	targeting synaptic vesicle proteins SV2A; target has no reference region	binds specifically to the synaptic vesicle glycoprotein 2A	lower sensitivity compared to [11C]-UCB-J	[276], [277]
[18F]FIMX	PD, addiction, epilepsy, neuropathic pain, and depression	targeting metabotropic glutamate receptors mGluR1	very good in vivo block response; fast kinetics	[272]
[18F]JNJ-64413739	purinoceptor P2X7	N.A.	first P2X7-type of tracer; no suitable reference region	[272]
[18F]BCPP-EF	targeting mitochondrial complexes MC1	quantitative imaging of MC-1 activity in the living brain	high brain uptake, suitable kinetics, large dynamic range in vitro	[301-307]
[18F]MNI-654 [18F]MNI-659	AD, HD	characterizing adenosine receptors across multiple regions of the brain	suitable pharmacokinetics; significant response in HD	[311]
[64Cu]Cu-SARTATE [68Ga]DOTA-TOC [68Ga]Ga-DOTA-NOC [68Ga]Ga-NODAGA-JR11 [68Ga]Ga-DOTA-TATE	neuroendocrine tumors;	targeting somatostatin receptor 2	peptide vectors	[4], [180], [272]

N.A. not applicable or not available.

Table S3. PET Radiopharmaceuticals used in cardiology (references over the last ten years)

Radiotracer	Disease	Molecular target	Function	Properties	Ref.
[82Rb]chloride	cardiac conditions	cardiac tissue	diagnostic; monitoring the cardiac flow	capacity to accurately quantify MBF and a low delivered radiation exposure for a rest/stress test	[344]
[15O]H_2O	myocardial perfusion,cerebral and tumor perfusion	N.A.	tracer for quantitative measurement of cerebral blood flow	the short half-life of ^{15}O results in the challenges in clinical use	[345], [358], [359]
[13N]NH_3	cardiovascular events, PC and encephalopathy	myocardial tissue, liver, kidneys and	imaging agent for assessing regional blood flow in tissues; for elucidation of NH3 metabolism in patients with hepatic	ammonia N13 enters the myocardium through the coronary arteries; well-validated radiotracer for clinical management; it is also used in PC due to	[329],[332-334],

		brain	encephalopathy; potentially a tumor imaging agent	the up-regulation of NH3 during glutamine synthesis in tumors	[357]
[^{15}O]CO	cardiovascular events	myocardial tissue	myocardial function	the most common tracers used for non-invasively measuring oxygen consumption and blood volume	[344]
[^{18}F]flurpiridaz	myocardial perfusion	mitochondrial complex I	diagnostic/imaging	novel PET tracer	[342], [343], [363-366]
[^{18}F]FBnTP		mitochondrial membrane		rapid myocardial uptake and retention and high myocardium/liver, myocardium/blood and myocardium/lung contrast in animal studies; few human studies reported to date	[360]
[^{18}F]FTPP		mitochondrial membrane			[360], [374]
[^{18}F]FDHR		mitochondrial complex I			[360], [370]
[^{18}F]FDM	atherosclerosis	mannose receptors	progressive inflammation in atherosclerotic plaques	it is (mannose) an isomer of glucose that is taken up by macrophages through glucose transporters;	[374]
[^{18}F]macroflor	atherosclerosis	macrophage-targeted polyglucose nanoparticle	immunoimaging; nanoparticle uptake	noninvasive assessment of the immune system in atherosclerosis;	[375], [402]
[^{64}Cu]DOTA-ECL1i	lung inflammation	chemokine receptor type 2 (CCR2)	detection of CCR2-directed inflammation	sensitive and specific detection of CCR2+ cells	[381-384]
[^{64}Cu]DOTA-DAPTA-comb	initiation and progression of atherosclerosis	chemokine receptor CCR5	specific imaging of CCR5	nanomedicinal approach towards cardiovascular diseases	[385]
[^{68}Ga]DOTATATE/DOTANOC	inflammatory conditions related to plaques	N.A.	functional imaging of plaques	increased uptakes in coronary arteries and large arteries; comparable diagnostic accuracy	[387], [388-392]

Table S4. PET Radiopharmaceuticals used in bacteria imaging (references over the last ten years)

Radiotracer	Type of bacteria	Properties	Ref.
* [^{18}F]FHM (maltohexaose)	*S. aureus*	better than FDG in differentiating non-infection inflammation from infection	[413]
*[^{18}F]FDS (sorbitol)	*K. pneumoniae*	better than FDG to detect lung infection from inflammation	[423]

*[^{18}F]maltotriose	N.A.	imaging bacterial infections in animals; future applications in clinics	[428]
*[^{18}F]FDS	*E.coli* *Enterobacteriaceae*	diagnosis and monitoring therapy; diagnostic for infections	[422-424]
*[^{18}F]FDG-6-P	*S.aureus*	potential to differentiate infection from inflammation	[419]
*[^{18}F]isonicotinic acid	*M.tuberculosis*	non-invasive approach to localize infectious foci; tested only on mice	[413]
*[^{18}F]FIAU	*E.coli* *P.aeruginosa*	engineered pathogens for evaluating experimental therapeutics	[413]
*[^{18}F]PABA	*S.aureus*	non-invasive tool for detecting/localizing/monitoring infections	[413]
*[^{68}Ga]TAFC *[^{68}Ga]FOXE	*A.fumigatus*	very promising for detection of infections with high sensitivity	[413], [420]
[^{64}Cu]ProT (prothrombin)	*S.aureus*	non-invasive detection with an analog of ProT	[413]
[^{64}Cu]JF5 mAb	*A.fumigatus*	localized aspergillus infection	
[^{18}F]maltose	*E.coli*	identifying drug resistance; promising for bacterial infection imaging	[426]
[^{18}F]trimethoprim	*E.coli* *P.aeruginosa* *S.aureus*	infection imaging	[427]
[^{68}Ga]UBI-29-41	*S.aureus*	non-toxic, identifies infectious foci in humans; correlated with degree of infection need further studies;	[413-417]
[^{68}Ga]UBI-31-38	*S.aureus*	good localization of infection site; promising results in humans	[414-417]
[^{68}Ga]TBIA101 (depsidomycin derivative)	*M. tuberculosis* *S.aureus*	imaging inflammation but not necessarily infection; non-specific	[430], [431]
[^{124}I]FIAU (fialuridine)	*S.aureus*	well tolerated but of limited value for detection of prosthetic joint infection; low image quality/specificity	[418]
[^{11}C]PABA (para-aminobenzoic acid)	*E.coli*	imaging living bacteria in humans	[413]

* novel tracers
N.A. not applicable or not available.

Table S5. The most promising SPECT radiopharmaceuticals that were evaluated in preclinical or clinical trials in the last decade.

SSTR and PSMA targeting radiopharmaceuticals have been the focus of research in the past decade. The versatility of technetium chemistry is seen in the variety of bifunctional chelators and technetium metal fragments used in synthesizing promising compounds for SPECT imaging of neuroendocrine tumors and prostate cancer.

Radiotracer	Disease	Molecular Target	Properties	Ref.
**$[^{99m}$Tc(I)] NOTA / NODAGA – sst2 - ANT**	neuroendocrine tumors	somatostatin receptors	$[^{99m}$Tc(CO)3$]^+$ core<br>NOTA / NODAGA bifunctional chelators<br>sst2-ANT – somatostatin receptor antagonist	[512], [513]
**$[^{99m}$Tc(V)]N4 – SS – 01**	neuroendocrine tumors	somatostatin receptors	*trans*-[O = Tc = O]$^+$ core<br>[N4] – tetramino, tetradentate chelator<br>SS – 01 somatostatin receptor sst2 antagonist	[514]
**$[^{99m}$Tc(V)]TECANT - 1**	neuroendocrine tumors	somatostatin receptors	*trans*-[O = Tc = O]$^+$ core<br>[N4] – tetramino, tetradentate chelator<br>LM3 - somatostatin receptor sst2 antagonis	[515]
**$[^{99m}$Tc(V)]EDDA / HYNIC – Ahx – SS - 01**	neuroendocrine tumors	somatostatin receptors	$[^{99m}$Tc]-HYNIC core with EDDA as coligand<br>Ahx – spacer<br>SS – 01 somatostatin receptor sst2 antagonist<br>The presence of the spacer greatly increased uptake	[516]
**$[^{99m}$Tc(I)]MIP – 1404**	prostate cancer	PSMA	$[^{99m}$Tc(CO)3$]^+$ core<br>TIM chelator<br>Glutamate-urea-glutamate pharmacophore	[522], [523]
**$[^{99m}$Tc(V)]EDDA / HYNIC – iPSMA**	prostate cancer	PSMA	$[^{99m}$Tc]-HYNIC core with EDDA as coligand<br>Lys(NaI)-urea-Glu pharmacophore	[525 – 528]
**$[^{99m}$Tc(V)]O – N3S – PSMA – I&S**	prostate cancer	PSMA	[Tc≡O]$^{3+}$ core<br>Mercaptoacetyl-triserine chelator<br>Lys-urea-Glu pharmacophore	[529], [530]

			Found useful applications in radio-guided surgery.	
**[^{99m}Tc(V)]HYNIC – ALUG**	prostate cancer	PSMA	[^{99m}Tc]-HYNIC core with EDDA as coligand<br>6-hydrazinonicotinate – aminocaproic acid-lysine-urea-glutamate pharmacophore	[524]
**[^{123}I]MIP – 1074**	prostate cancer	PSMA	glutamate-urea heterodimer that inhibits the enzymatic activity of PSMA<br>high affinity and specificity<br>internalizes in prostate cancer cells that express PSMA	[519 – 521]
**[^{99m}Tc(V)]maraciclatide**	breast, lung, prostate cancer	$\alpha\nu\beta3$ integrin	Cyclic, synthetic peptide containing the RGD motif, with high affinity for the $\alpha\nu\beta3$ integrin	[533]
**[^{99m}Tc(V)]3P4-RGD2**	breast, lung, esophageal cancer	$\alpha\nu\beta3$ integrin	Cyclic RGD dimer. The compound contains the PEG4 linker in order to improve the tumor uptake of the compound	[534 – 540]
**[^{99m}Tc(III)]3SPboroxime**	various heart conditions	passive diffusion	Member of the BATO (boronic acid adducts of technetium dioximes) class of [^{99m}Tc](III) complexes.<br>Isostructural derivative of [^{99m}Tc]-teboroxime, where the methyl group appended to the boronate cap was replaced by a sulfonyl group.<br>The new compound had similar initial heart uptake and delayed myocardial washout resulting in more favorable imaging properties.	[551 – 554]
**[^{99m}Tc(V)]N – MPO**	various heart conditions	passive diffusion	[^{99m}Tc]N core was coordinated by the 2-mercaptopyridine oxide bidenate ligand in combination with biphosphine. The compound shows similar pharmacokinetic properties to currently used radiopharmaceuticals, but has better heart to liver ratio.	[546]
**[^{99m}Tc(I)]benzothiazole derivatives**	AD	β – amyloid plaques	Obtained by the replacement of the phenyl group in 2 – phenylbenzothiazole with the cyclopentadienyl tricarbonyl	[560]

			Cp[^{99m}Tc](CO)3 core. The compounds show impressive brain uptake and maintain the Aβ binding affinity of the 2-Arylbenzothiazole scaffold.	
**[^{111}In]DTPA-RP782**	atherosclerosis, inflammation	matrix metalloproteinase inhibitors	Could be used for molecular imaging of atherosclerosis, aneurysm, and monitoring distinctive MMP activities. Peptidomimetic vector; further pre-clinical and clinical studies are mandatory to assess its feasibility	[406-409]

Table S6. Chemical structures of some of the most representative ligands and chelating agents for PET and/or SPECT radiopharmaceuticals.

PET		
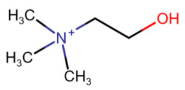<br>**Choline**	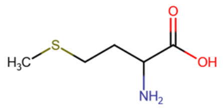<br>**Methionine**	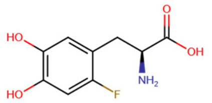<br>**FDOPA**
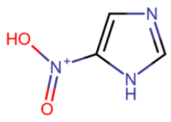<br>**4-nitromidazole**	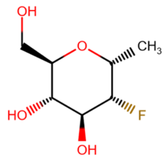<br>**FDG**	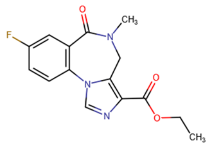<br>**Flumazenil**
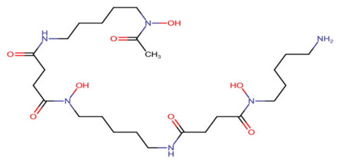<br>**Desferrioxamine B**	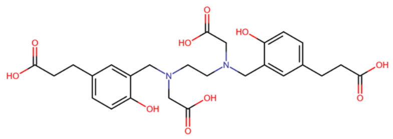<br>**HBED-CC**	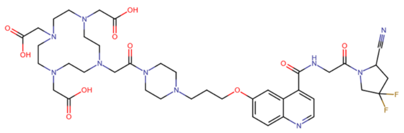<br>**FAPI-04**
PET and SPECT		

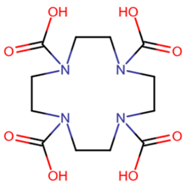<br>**DOTA**	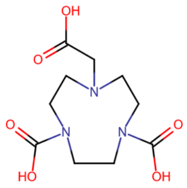<br>**NOTA**	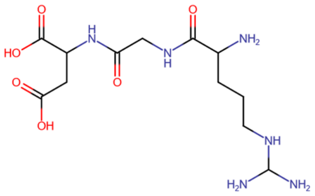<br>**RGD motif**
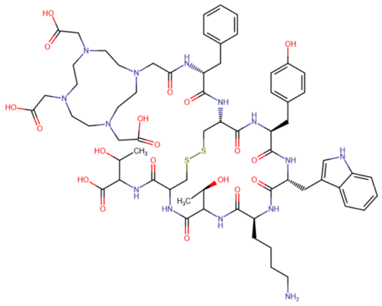<br>**DOTATATE**		
SPECT		
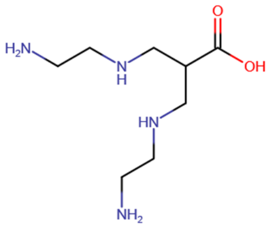<br>**6-carboxy- 1,4,8,11 – tetraazaun-decane (N4)**	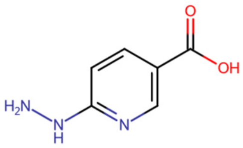<br>**Hydrazinonicotinamide acid**	
